# Design, Synthesis and Biological Evaluation of Ketoprofen Conjugated To RGD/NGR for Targeted Cancer Therapy

**Published:** 2018

**Authors:** Bahareh Shokri, Afshin Zarghi, Soraya Shahhoseini, Reza Mohammadi, Farzad Kobarfard

**Affiliations:** *Department of Medicinal Chemistry, School of Pharmacy, Shahid Beheshti University of Medical Sciences, Tehran, Iran.*

**Keywords:** RGD, NGR, Tumour targeting, Integrin, Aminopeptidase N

## Abstract

It is well known that Arginine-Glycine-Aspartic acid (RGD) and Asparagine-Glycine-Arginine (NGR) peptides preferentially bind to integrin receptors and aminopeptidase N respectively and these two receptors play important roles in angiogenesis. Therefore ketoprofen as a non-selective cox Inhibitor was conjugated with linear RGD and NGR to take advantage of targeting capability of these two motifs and delivering ketoprofen to these cancer cells with overexpression of integrin and aminopeptidase N. In order to investigate the impact of possible steric hindrance due to the attachment of the drug to the peptide, a linear six carbon (hexanoic acid) linker was also used as a spacer. Cytotoxic effect of the synthesized compounds was evaluated against a group of cancer cell lines, including MCF-7, A2780 (α_v_β_3_ positive), OVCAR3 (high α_v_β_3_), HT-1-80 (high CD13) and SKOV-3 (CD13 positive). Both NGR and RGD conjugated forms of ketoprofen showed higher cytotoxic activity against OVCAR3 and HT-1-80 respectively.

## Introduction

Cancer is unusual cell growth and division which can spread to different parts of body. Chemotherapy is a main approach for cancer treatment and limited by low selectivity of chemotherapy drugs for normal tissues, especially tissues with rapidly cells division and growth.

Angiogenesis is crucial and essential factor in the invasive growing and proliferation of cancer cells. It is the process of formation of new blood vessel from pre-exciting vessel which includes the migration, growth, and differentiation of endothelial cells, inside the walls of blood vessel (1). Integrin as a cell surface and adhesion receptor interacts to ECM proteins. They are heterodimer glycoprotein receptors and have 24 pairs α and 8 β subunits and have key roles in cell growing, proliferation, migration, differentiation and cell survival signaling (2). α_v _integrin is highly overexpressed on activated endothelial of cancer cells and tumor cells, but it is not expressed in resting endothelial cells and most normal organ systems, making it a potential target for antiangiogenic strategy in cancer therapy (3).

Integrins are ideal pharmacological targets based on their key role in angiogenesis and tumor development and on their easy accessibility as cell surface receptors interacting with extracellular ligands. α_v_β_3_ was the first vascular integrin, targeted to suppress tumor and ischemia-induced angiogenesis. Antagonists of integrin α_v_β_3_ are currently tested in clinical trials as antiangiogenic gents (4). On the other hand NSAIDs have shown significant anti-proliferative effects through the inhibition of COX-2 (5, 6). It is also proven that the anti-proliferative activity of NSAIDs is via anti-angiogenesis activity of these drugs (7, 8) and inhibition of COX-2 by NSAIDs have suppressed tumor growth in animal models and reduced the risk of developing cancer in humans (6, 9 and 10). Moreover NSAIDs exert some of their anti-thrombotic and anti-inflammatory effects by interfering with integrin function. For example, indomethacin and aspirin inhibit platelet aggregation by suppressing activation of integrin α_v_β_3_, the main fibrinogen receptor in platelets (11). Addition, several other reports indicate that NSIADs inhibit α_v_β_3_ integrin, which affects the endothelial-cell spreading, migration and angiogenesis processes (12). In light of the evidences which support the role of NSAIDs in cancer cells suppression and considering the overexpression of COX-2 enzyme in cancer cells (6, 9 and 10), targeted delivery of these therapeutic agents to the cancerous cells could maximize their efficiency and minimize their side effects. Nemours studies in tumor targeting have been implemented by using certain peptide sequences which are conjugated to the drug of interest and this strategy has been recently introduced as one of the useful strategies in medicinal chemistry of chemotherapeutic agents.

The two tripeptidic sequences, RGD (Arginine-Glycine-Aspartic acid) and NGR (Asparagine-Glycine-Arginine) motifs have been recognized as targeting tools based on phage display studies (13). RGD is a well-known peptide sequence for targeting integrin receptors and can bind to a_v_b3 and a_v_b5 integrin receptors subunits which are overexpressed during the angiogenesis process of cancer cells (14). Since α_v _integrins are overexpressed on surface of cancer cells (15-19), RGD as an integrin ligand can be used as a targeting system for delivering chemotherapeutic agents to the cancer cells (3). For example, RGD has receptor on activated platelet; Shaoming Jin has used aspirin conjugated with RGDV for delivery of Aspirin to thrombus (20). NGR peptide has also been used as targeting tool for aminopeptidase N (CD13) which is overexpressed in cancer and inflammatory diseases, 

Ketoprofen is a nonsteroidal anti-inflammatory drug (NSAID) which inhibits COX-1 and COX-2 enzymes. Numerous studies demonstrate that NSAIDs have anticancer properties and reduced the risk of cancers like colon and liver (21) Several structural modifications have been made in NSAIDs to change their properties such as lipophilicity, solubility in water and side effects (22-24). Cheng *et al*. have demonstrated that ketoprofen inhibits human colon tumor cell N-acetyltransferase (NAT) activity, gene expression and DNA adduct formation for the first time in 2006 (25). Marjanovic and his coworkers have also reported potential antitumor activity for the amide derivatives of ketoprofen and fenoprofen later in 2007 (26). Based on the above mentioned fact, we decided to prepare RGD and NGR conjugates of ketoprofen and evaluate their anti-cancer activity on a selected group of cancer cell lines. The conjugated forms are expected to be delivered rather selectively to tumor cell lines due to the overexpression of RGD and NGR receptors on the surface of these cells. In order to investigate the impact of possible steric hindrance due to the attachment of the drug to the peptide, a linear six carbon (hexanoic acid) linker was also used as a spacer.

## Experimental

*Materials and methods*

All chemicals and solvents were purchased from Merck (Darmstadt, Germany). N^a^-9-Fluorenylmethoxycarbonyl (Fmoc)-protected amino acids, Rink amid resin, 1-[Bis(dimethylamino)methylene]-1*H*- 1,2,3-triazolo[4,5-*b*] pyridinium 3-oxid hexafluorophosphate (HATU) were purchased from GL Biochem Company (Shanghi) Ltd. ^1^HNMR spectra were recorded on a 500 MHz Bruker spectrometer. ESI-MS spectra were obtained by Agilent 6410 Triple Quad LC/MS. IR spectra were recorded on a PerkinElmer IR spectrophotometer as potassium bromide discs. The purity of compounds was confirmed by thin layer chromatography using Whatman Sil 

G/UV254 silica gel plates as the stationary phase and with suitable mobile phase with fluorescent indicator, and the spots were visualized under 254 and 366 nm illumination. All products was purified by reverse phase HPLC on a Knaur chromatography system equipped with a Waters Prep Nova-Pak^®^ HR ODS column (250 × 4.6 mm; porosity 5 μm).

*General procedure for synthesis RGD conjugated with ketoprofen*

Rink amide resin (0.5 g) was placed in a reactor and suspended in DMF under nitrogen atmosphere for 15 min. The fmoc group was removed with 20% piperidine in DMF (3 × 5 mL). After washing with DMF, coupling reaction was conducted. For each amino acid coupling: 2eq AA, 2eq HATU, 4eq N,N-diisopropylethylamine (DIEA), 15 mL DMF was mixed and added to the resin. The mixture was purged with N_2_ gas for 20 min. The completion of each coupling reaction and fmoc deprotection was tested by Kaiser Test which is the reaction of ninhydrin with amines. 

After the last amino acid coupling reaction and removal of fmoc protecting group, the ketoprofen was coupled to the free amino group of the peptide, using the same standard procedure which is used for the coupling of amino acids. Finally resin washed and dried then linear peptide cleaved with trifluoroacetic acid (TFA) cocktail: TFA: Thioanisol: p-cresol: triisopropylsilane (TIS) (84%: 12%: 2%: 2%), during 2 h at room temperature. The reaction mixture was filtered and cold Diethyl Ether (50 mL) was added to the filtrate. The precipitate thus obtained was separated using centrifugation.

*RGD*

The title compound (50 mg) was obtained as a white solid with 63% yield at 97% purity (based on chromatography, mobile phase 30% CH_3_CN in H_2_O+ 0.1% TFA); 1H NMR (500 MHz, DMSO-*d6*) *δ*: 1.54 (s, 2 H, CH_2_), 1.70 (s, 2H, CH_2_), 2.63-2.69 (m, 2H, CH_2_), 3.11 (s, 2H, CH_2_), 3.86 (s, 3H, CH_2, _CH), 4.50-4.53 (quartet, J = 0.01, 1H, CH), 7.15-8.68 ppm (10H, NH, NH_2_); IR (KBr): υ (cm-1) = 1656, 3330; LC-MS (ESI) m/z (M + H)^+^ calcd for C_12_H_23_N_7_O_5_: 346.0, found: 346.1. 

*NGR*

The title compound (57 mg) was obtained as a white solid with 64% yield at 93% purity (based on chromatography, mobile phase 30% CH_3_CN in H_2_O+ 0.1% TFA); 1H NMR (500 MHz, CDCl_3_) *δ*: 1.50 (s, 2 H, CH_2_), 1.99 (s, 2H, CH_2_), 2.5 (s, 1H, NH), 2.58-2.69 (m, 2H, CH_2_), 3.23 (s, 2H, CH_2_), 4.03 (s, 2H, CH_2_), 7.15-8.3 ppm (10H, NH, NH_2_); IR (KBr): υ (cm-1) = 1656, 3330; LC-MS (ESI) m/z (M + H)^+^ calcd for C_12_H_23_N_7_O_5_: 346.0, found: 346.1.

*Keto-RGD*

The title compound (83 mg) was obtained as a white solid with yield 65%, 98% purity (based on chromatography, mobile phase 30% CH_3_CN in H_2_O+ 0.1% TFA); 1H NMR (500 MHz, DMSO-*d6*) *δ*: 1.32-1.33(d, J = 0.01, 3H, CH_3_), 1.45-1.46 (m, 2H, CH_2_), 1.64-1.82 (m, 2H, CH_2_), 2.5 (s, 1H, NH), 2.96,3.07 (m, 2H, CH_2_), 3.64-3.69 (m, 2H,CH_2_), 3.82-3.85 (m, 1H, CH), 4.15-4.16 (m, 2H, CH_2_), 4.26-4.27 (m, 1H, CH), 4.38-4.43 (m, 1H,CH_1_), 7.05-8.31(15H, NH, NH_2_ and aromatic hydrogen), 11.8 (br s, 1H, OH) ppm; IR (KBr): υ (cm-1) 1656, 3330 ; LC-MS (ESI) m/z (M + H)^+^ calcd for C_28_H_35_N_7_O_7_: 581, found: 582.2. 

*Keto-NH-(CH*_2_ *)*_4_ *-CO-RGD*

The title compound (103 mg) was obtained as a white solid with yield 65%, 98% purity (based on chromatography, mobile phase 30% CH_3_CN in H_2_O+ 0.1% TFA); 1H NMR (500 MHz, CDCl_3_) *δ*: 1.05 (s, 3H, CH_3_), 1.20-1.23 (m, 2H, CH_2_), 1.28 (s, 2H, CH_2_), 1.48 (m, 4H, CH_2_), 1.7 (m, 2H, CH_2_), 2.07 (m ,2H, CH_2_), 2.8 (br s, 2H, CH_2_), 3.17 (m, 2H, CH_2_), 3.38 (m, 2H, CH_2_), 3.46-3.52 (q, J = 0.02, 1H, CH), 4.16 (s, 2H, CH_2_), 4.55 (s, H, CH), 5.88-5.93 (m, H, CH), 7.00-7.8 ppm (18H, NH, NH_2 _and aromatic hydrogen), IR (KBr): υ (cm-1) 1654, 3356 ; LC-MS (ESI) m/z (M + H)^+^ calcd for C_34_H_47_N_9_O_7_: 693, found: 694.2.

*Keto-NGR*

The title compound (73 mg) was obtained as a white solid with yield 57%, 98% purity (based on chromatography, mobile phase 30% CH_3_CN in H_2_O+ 0.1% TFA); 1H NMR (500 MHz, CDCl_3_) *δ*: 1.05 (s, 3H, CH_3_), 1.25 (m, 2H, CH_2_), 1.45 (m, 2H, CH_2_), 2.5 (s, 1H, NH), 2. 6 (m, 2H, CH_2_), 3.47 (m, 2H,CH_2_), 3.6 (m, 1H, CH), 3.9 (m, 2H, CH_2_), 4.16 (m, 1H, CH), 4.43 (m, 1H,CH), 7.05-8.29 (19H, NH, NH_2_ and aromatic hydrogen), 11.8 (br s, 1H, OH) ppm; IR (KBr): υ (cm-1) 1656, 3330; LC-MS (ESI) m/z (M + H)^+^ calcd for C_28_H_36_N_8_O_7_: 581, found: 582.2. 

*Keto-NH-(CH*_2_ *)*_4_ *-CO-NGR*

The title compound (110 mg) was obtained as a white solid with yield 69%, 97% purity (based on chromatography, mobile phase 30% CH_3_CN in H_2_O+ 0.1% TFA); 1H NMR (500 MHz, CDCl_3_) *δ*: 1.05 (d, 3H, CH_3_), 1.19-1.25 (m, 6H, 3CH_2_), 1.46-1.48 (m, 2H, CH_2_), 1.64 (m, 2H, CH_2_), 2.28 (br s, 2H, CH_2_), 2.49 (s, 1H , NH), 2.90 (br s, 2H, CH_2_), 2.97 (s, 2H, CH_2_), 3.40 (br s, 2H, CH_2_), 3.46-3.51(q, H, CH), 4.12 (s, 2H, CH_2_), 4.8 (m, 1H, CH) 5.88-5.93 (m, 1H, CH), 7.00-8.03 ppm (20, H NH, NH_2, _aromatic ring); IR (KBr): υ (cm-1); IR (KBr): υ (cm-1) 1659, 1647, 3331; LC-MS (ESI) m/z (M + H)^+^ calcd for C_34_H_47_N_9_O_7_: 693, found 694.1. 

*Cell cytotoxicity*

In order to evaluate the cytotoxic activity of the synthesis compounds, five cell lines: A2780 (low α_V_β_3_), OVCAR-3 (high α_V_β_3_), SKOV-3 (low CD13), HT-1080 (high CD13) and MCF-7 and fibroblast cell were selected. A2780 and MCF-7 are both cancer cells, but A2780 is α_v_ β3 integrin overexpressing cell line. Three concentrations of products were prepared and were evaluated after 48 h by using MTT method. Therefore cells cultured in culture media (RPMI1640) at 37 °C under 5% CO2 supplemented with 10% fetal bovine serum (FBS), 100 U/mL penicillin and 100 μg/mL streptomycin. Nearly 10^4^ cells were seeded into 96-well plates and were grown for 24 h in incubator. Compounds and doxorubicin added to wells, cells were incubated at 37 °C for 48 h. Cells without any adding of compound were used as negative control, Doxorubicin was used as positive control and DMSO as the solvent of the test compounds. After 48 h, 10 μL of MTT (5 mg/mL in PBS) was added at 37 °C for 4 h. The medium with MTT was removed and 100 μL DMSO was added to each well, cover the plate from light, shake it for 5 min, then optical densities were read at 570 nm with a reference wavelength of 630 nm as background using a spectrophotometer plate reader (Infinite® M200, TECAN).

*Molecular modeling (docking) studies*

Docking studies were performed using AutoDock Vina software for all the synthesized compounds to study their interactions with extracellular domain of the α_v_β3 integrin receptor in the presence of the Mn^2+^ ion and with active sites of APN. The crystal structure of the extracellular domain of the α_v_β3 integrin receptor in the presence of the Mn^2+^ ion (PDB entry code: 1L5G) (27) was obtained from the RCSB Protein Data Bank. Since in the X-ray structure the head group of α_v_β_3_ integrin, which comprises the β propeller domain of α_v_ and the β_3_ domain of β_3_, has been identified as the ligand binding region, only the globular head is considered for docking study. The X-ray crystal structure of APN was obtained from the RCSB Protein Data Bank (PDB code: 2DQM) (28).

All the compounds were built using hyperchem version 8 and subsequently minimized. The protein structure was prepared for docking using AutoDock Tool. Polar hydrogen was added and non-polar hydrogen was merged and finally Kollman united atom charge and atom type parameters were added to 1L5G for RGD products and 2DQM for NGR products. Grid map dimensions for 1L5G (18.6378 × 41.9200 × 43.1259) were set surrounding active site. The energy minimized ligands were docked in binding site of α_v_β_3_ integrin receptor. The quality of the docked structures was assessed by measuring the intermolecular energy of the ligand-enzyme assembly. Grid map dimensions for 2DQM (71.9186 × 47.8031 × 7.3077) were set surrounding active site. The energy minimized ligands were docked in binding site of APN.

## Results and Discussion

*Chemistry*

**Scheme 1:**
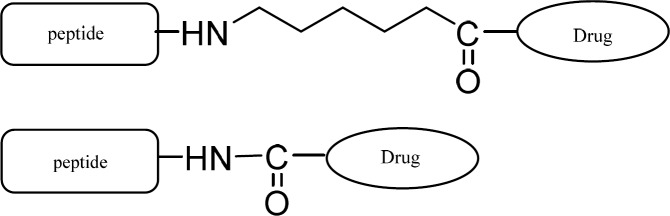
Peptide-drug and peptide-spacer-drug conjugates

**Scheme 2:**
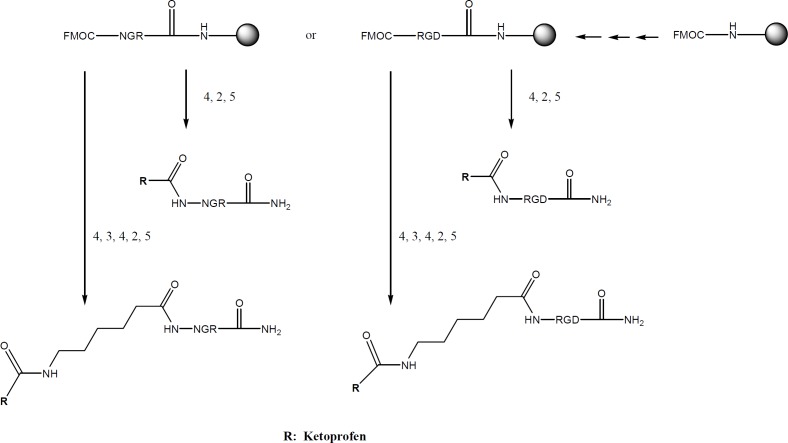
Solid-phase peptide synthesis of RGD or NGR Drug conjugated, (1) solid phase peptide synthesis, (2) ketoprofen, HATU, DIEA, DMF, (3) fmoc-amino hexanoic acid, HATU, DIEA, DMF, (4) fmoc deprotection by Piperidine 20%, (5) cleavage by TFA, Thioanisol, *p*-cresol, TIS

**Scheme 3:**
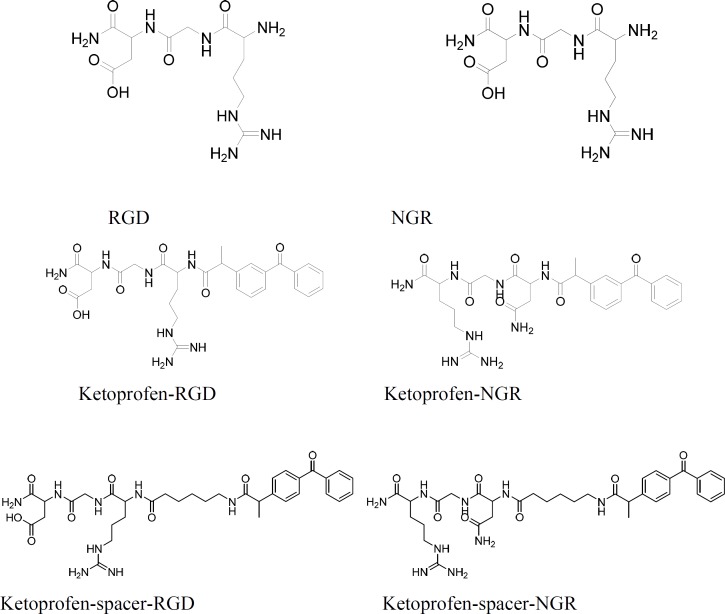
Structure of all compounds.

Ketoprofen were selected as NSAIDs and fmoc peptide synthesis strategy was used to make both the peptide-drug conjugates and peptide-spacer-drug conjugates. Therefore, fmoc-6-amino hexanoic acid was needed to make the peptide-spacer-drug conjugates (Scheme 1). The synthesis process is summarized in Scheme 2. The structures of all products are showed in Scheme 3.

Peptide = RGD or NGR, Drug = ketoprofen

*Biological activity*

RGD and NGR are promising peptide sequences for targeted drug delivery to tumor cells. They can carry their drug conjugated to their target tumor. Ketoprofen was attached to N-terminus of the tripeptides RGD and NGR through its carboxylic acid group and thus an amide bond is responsible for the conjugation. In order to avoid the possible steric hindrance which is caused by the closeness of ketoprofen to the tripeptide, another from of the conjugate was also synthesized in which ketoprofen was attached to the tripeptide through a linear six carbon linker. Anti-proliferation activities of the synthesized compound were evaluated on a selected group of cancer cell lines. The results are summarized in Table 1: comparing the results reveals the following points: 

a. No inhibitory activity was observed on MCF-7 (as a tumor cell without over-expressed RGD- or NGR-binding protein on its surface) or Fibroblasts (as normal non-tumor cells).

b. ketoprofen caused no inhibition at 100 µm concentration either on A2780 or on OVCAR-3. However the conjugated forms of ketoprofen with RGD either with or without spacer, showed >50% inhibition on both cell lines. The two cell lines are well recognized for the overexpression of RGD receptors (A2780 with low and OVCAR-3 with high expression of α_v_β_3_) on their surface. In case of A2780, keto-spacer-RGD showed higher inhibitory activity than keto-RGD (63 *vs.* 55% inhibition) which indicates possible role of spacer either in better binding to the receptor or in mitigating the steric hindrance for better binding of RGD to the cell surface. Surprisingly, the opposite pattern is observed in case of OVCAR-3 which shows the possible impediment of linker group for the ligand-receptor binding. 

c. NGR conjugates of ketoprofen caused 3-7 times higher inhibition compared to ketoprofen on HT-1080 and SKOV-3. These are the cancer cell lines with over expression of NGR receptor (SKOV-3 with low and HT-1080 with high expression of CD13) on their surface. Therefore it could be speculated that the NGR companionship with ketoprofen has improved its cytotoxic activity on these cell lines. In both cases, the conjugated form which contains the spacer between ketoprofen and NGR showed higher activity which could be assigned to the less hindrance for ligand- receptor binding.

**Table 1:** Inhibitory % values of compounds at 100 µM concentration

	**A2780**	**OVCAR3**	**HT-1080**	**SKOV-3**	**MCF-7** *	**Fibroblast**
Ketoprofen	No inhibition	No inhibition	9.25 ± 1.3	14 ± 4.7	-	No inhibition
Keto-RGD	55 ± 0.5	77 ± 0.47	-	-	13 ± 0.2	No inhibition
Keto-NH-(CH_2_)_4_-CO-RGD	63 ± 0.6	65 ± 2.7	-	-	36 ± 0.5	No inhibition
Keto-NGR	28 ± 2.6	-	54 ± 0.5	50 ± 0.6	-	No inhibition
Keto-NH-(CH_2_)_4_-CO-NGR	41 ± 0	-	76 ± 1	60 ± 0.2	-	No inhibition
RGD	10 ± 2.2	No inhibition	No inhibition	9.5 ± 0.5	No inhibition	No inhibition
NGR	6 ± 1.5	No inhibition	6.5 ± 0.5	No inhibition	No inhibition	No inhibition
Doxorubicin**	65 ± 1.2	45.25 ± 1.25	26.7 ± 4.8	29.5 ± 2.5	38 ± 0.53**	-

* : at 50 µM.

** : at 5 µM.

*Molecular modeling (docking) studies*

**Figure 1:**
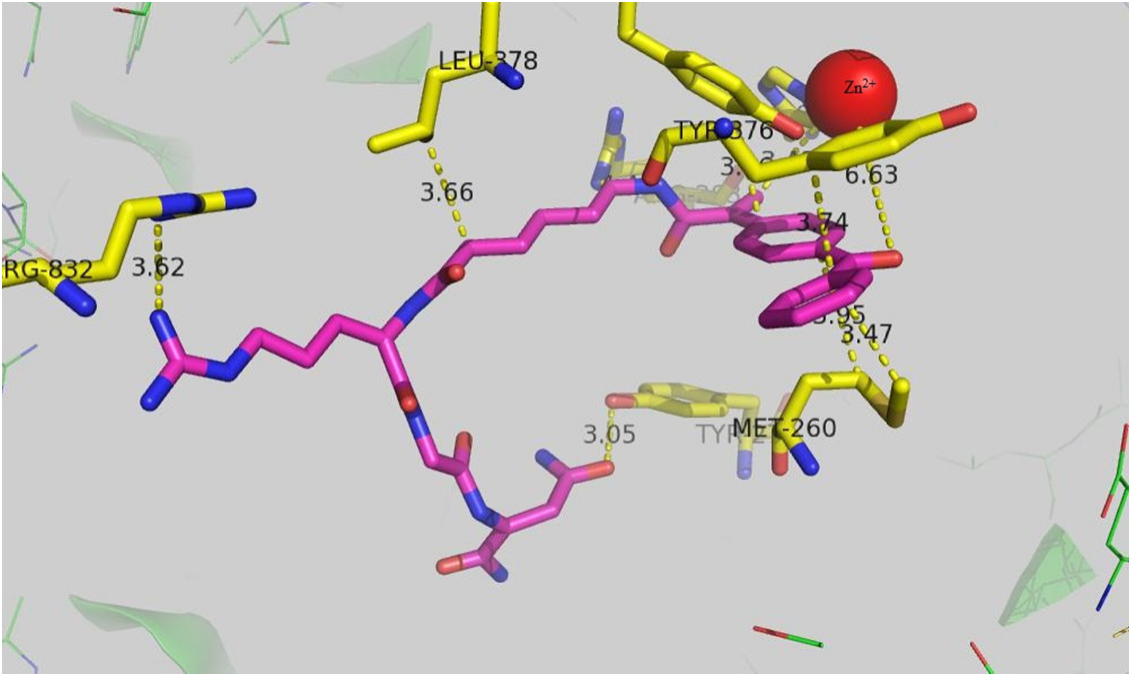
The docking result of ketoprofen-spacer-NGR with aminopeptidase N (PDB code: 2DQM)

Aminopeptidase N (APN/CD13) which is assumed to be the target of ketoprofen-NGR conjugate is a zinc-dependent metalloprotease which is overexpressed in many disease such as cancer and inflammation. Bestatin is the first marketed APN inhibitor which has been introduced in 1976 (29). Therefor a docking study was conducted to investigate the possible binding mode of ketoprofen-spacer-NGR which has highest activity against HT-1080 cells. The result is presented in Figure 1. The carbonyl oxygen of ketoprofen can interact with the zinc ion with the distance of ca 6.6A˚. The distance of the guanidine residue of Arg 832 with guanidine residue of keto-spacer-RGD was 3.62A˚ and they form hyrogen bond. Tyrosine 376 is in proximity of the ketoprofen ring, suggesting a π-π stacking interaction between the two rings with the distance of 3.66A˚. Leucin 378 residue has come into contact with the 6 membered spacer which was used to prevent the possible steric interference between ketoprofen and NGR and thus causing the more efficient binding of ketoprofen-spacer-NGR to the receptor.

**Figure 2:**
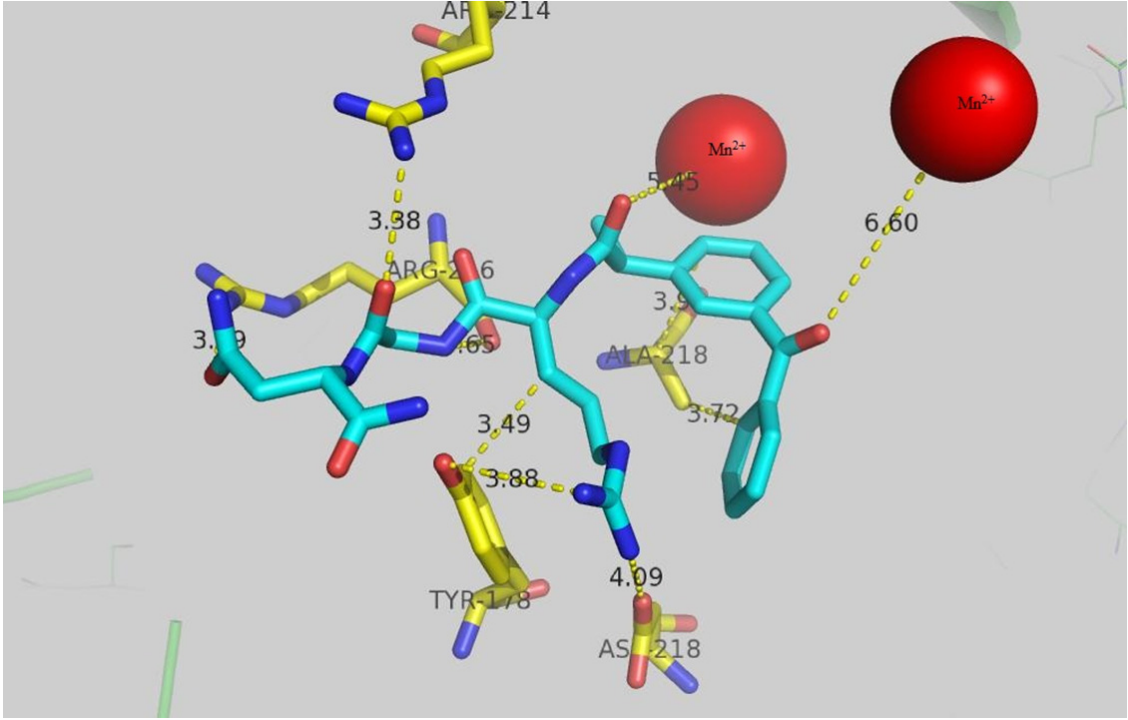
The docking result of ketoprofen-spacer-RGD with α_v_ β_3 _integrin (PDB code: 1L5G)

RGD target α_v_β_3 _integrin subunit that is overexpressed in cancer. In order to explore the possible mode of interaction for keto-RGD conjugate, docking study was carried out using 1L5G crystallography of α_v_β_3 _integrin and the results are presented in Figure 2. Arg 216 in β chain forms hydrogen bond with gunidine residue with the distance of 3.29 A˚and also (β)-Arg 214 forms hydrogen bon with 3.38A˚ from NH_2_ of asparagin moiety. The distance of Mn^2+ ^ion in active site of enzyme from the carbonyl group of ketoprofen is 6.6A˚. Another Mn^2+ ^ion in active site has 5.45A˚ distance from the carbonyl group of RGD peptide chain. Hydrophobic residue of (β)-Ala 218 with the distance of 3.9A˚ and 3.7A˚ has come into contact with ketoprofen rings. Hydroxyl group of (α)-Tyr178 forms hydrogen bond with NH of guanidine in keto-RGD with the distance of 3.88A˚ and also tyrosin ring has 3.49A˚ distance from CH_2_ side chain of Arg in keto-RGD.

## Conclusion

RGD and NGR peptide sequences are capable of guiding a chemotherapeutic agent to its target. This strategy could be used for maximizing the efficiency of chemotherapy based on the fact that in many cancer cells, specific membrane receptors are overexpressed on the cell surface. The result of present study show that ketoprofen conjugated to RGD and NGR have higher cytotoxic activity compared to ketoprofen itself which strongly supports the hypothesis of targeting by peptide-drug conjugates.
